# Supplementary material for: Intrathecal Injection of Autologous Mesenchymal Stem-Cell-Derived Extracellular Vesicles in Spinal Cord Injury: A Feasibility Study in Pigs
Source: Int J Mol Sci. 2023 May 4;24(9):8240. doi: 10.3390/ijms24098240 (PMC10179045; doi:10.3390/ijms24098240)
Supplement: Supplementary file 1 [file ijms-24-08240-s001.zip › ijms-2331609-supplementary.pdf]

Table S1. Concentrations generated with the multiplex analysis of EVs and mesenchymal stem cells supernatant as a control. Mean values ± SD from experiments performed are shown. Bold text were p-values and calculated by unpaired t-test were < 0.01, *p< 0.001.

Cytokine	MSC supernatant, 10μg	EVs, 10μg
GM-CSF	0.02±0	**0.03±0***
IFN- g	1.80±0.53	**2.34±0.05***
IL-10	<0.01	0.02±0
IL-18	0.42±0.11	**1,56±0.08***
IL-1a	1.14±0.80	0.52±0.02
IL-1b	0.03±0	0.03±0
IL-1ra	0.015±0.01	0.01±0
IL-2	0.07±0.01	**0.016±0.01***
IL-4	0.015±0.01	**0.02±0***
IL-6	0.03±0	0.03±0
IL-8	3.08±0.90	**1.26±0.05**
TNF-α	0.04±0.01	0.016±0.005
